# Genetic dissection of Al tolerance QTLs in the maize genome by high density SNP scan

**DOI:** 10.1186/1471-2164-15-153

**Published:** 2014-02-24

**Authors:** Claudia T Guimaraes, Christiano C Simoes, Maria Marta Pastina, Lyza G Maron, Jurandir V Magalhaes, Renato CC Vasconcellos, Lauro JM Guimaraes, Ubiraci GP Lana, Carlos FS Tinoco, Roberto W Noda, Silvia N Jardim-Belicuas, Leon V Kochian, Vera MC Alves, Sidney N Parentoni

**Affiliations:** Nucleus of Applied Biology, Embrapa Maize and Sorghum, Road MG424, km 65, Sete Lagoas, MG 35701-970 Brazil; Instituto de Ciências Biológicas, Universidade Federal de Minas Gerais, Belo Horizonte, MG Brazil; Department of Plant Breeding and Genetics, Cornell University, Ithaca, NY USA; Departamento de Biologia, Centro Universitário de Sete Lagoas, Sete Lagoas, MG Brazil; Robert W. Holley Center for Agriculture and Health, U.S. Department of Agriculture – Agriculture Research Service, Cornell University, Ithaca, NY USA

**Keywords:** Genotyping-by-sequencing, MATE, Marker-assisted selection, Copy number variation

## Abstract

**Background:**

Aluminum (Al) toxicity is an important limitation to food security in tropical and subtropical regions. High Al saturation on acid soils limits root development, reducing water and nutrient uptake. In addition to naturally occurring acid soils, agricultural practices may decrease soil pH, leading to yield losses due to Al toxicity. Elucidating the genetic and molecular mechanisms underlying maize Al tolerance is expected to accelerate the development of Al-tolerant cultivars.

**Results:**

Five genomic regions were significantly associated with Al tolerance, using 54,455 SNP markers in a recombinant inbred line population derived from Cateto Al237. Candidate genes co-localized with Al tolerance QTLs were further investigated. Near-isogenic lines (NILs) developed for *ZmMATE2* were as Al-sensitive as the recurrent line, indicating that this candidate gene was not responsible for the Al tolerance QTL on chromosome 5, *qALT5*. However, *ZmNrat1*, a maize homolog to *OsNrat1*, which encodes an Al^3+^ specific transporter previously implicated in rice Al tolerance, was mapped at ~40 Mbp from *qALT5*. We demonstrate for the first time that *ZmNrat1* is preferentially expressed in maize root tips and is up-regulated by Al, similarly to *OsNrat1* in rice, suggesting a role of this gene in maize Al tolerance. The strongest-effect QTL was mapped on chromosome 6 (*qALT6*), within a 0.5 Mbp region where three copies of the Al tolerance gene, *ZmMATE1,* were found in tandem configuration. *qALT6* was shown to increase Al tolerance in maize; the *qALT6-*NILs carrying three copies of *ZmMATE1* exhibited a two-fold increase in Al tolerance, and higher expression of *ZmMATE1* compared to the Al sensitive recurrent parent. Interestingly, a new source of Al tolerance via *ZmMATE1* was identified in a Brazilian elite line that showed high expression of *ZmMATE1* but carries a single copy of *ZmMATE1*.

**Conclusions:**

High *ZmMATE1* expression, controlled either by three copies of the target gene or by an unknown molecular mechanism, is responsible for Al tolerance mediated by *qALT6*. As Al tolerant alleles at *qALT6* are rare in maize, marker-assisted introgression of this QTL is an important strategy to improve maize adaptation to acid soils worldwide.

**Electronic supplementary material:**

The online version of this article (doi:10.1186/1471-2164-15-153) contains supplementary material, which is available to authorized users.

## Background

Aluminum (Al) toxicity is one of the major constraints for crop production on acidic soils, which comprise over 50% of the world’s potentially arable lands [1]. Under low soil pH, Al is solubilized into highly rhizotoxic ionic forms, primarily Al^3+^, that inhibit root growth, reducing water and nutrient uptake [2]. Although liming and other agronomic practices can ameliorate soil acidity, such technologies are not readily available for a large number of small-scale farmers particularly in the developing world, where agriculture is the main source of income and food. In addition to naturally occurring acid soils, soil acidification has been reported after 21 years of no-tillage crop system in the south of Brazil [3], an agricultural production method now largely adopted worldwide [4]. Maize is the most produced cereal in the world and is widely used in animal feed, human food and industrial purposes. In addition, maize is a major staple food in Africa and Latin America where acid soils are common [5]. Therefore, efforts to elucidate the genetic and molecular mechanisms underlying maize Al tolerance are expected to accelerate the development of Al-tolerant cultivars.

Al tolerance has been shown to be controlled by major genes in wheat [6], sorghum [7, 8], and barley [9]. These plant species were used to clone the first Al tolerance genes, which were involved in organic acid exudation to exclude Al from the root apex. The Al tolerance gene in wheat encodes an aluminum-activated malate transporter (*TaALMT1*) responsible for malate release from root apices [6]. Subsequently, *ALMT1* homologs with a role in Al tolerance were identified in Arabidopsis [10], rape [11], and rye [12]. Another gene family involved in Al tolerance is the multidrug and toxic compound extrusion (MATE) family that was originally associated with Al-activated citrate release in sorghum (*SbMATE*) [8] and barley (aluminum-activated citrate transporter 1, *HvAACT1*) [9]. In addition, MATE homologs confer Al tolerance in Arabidopsis (*AtMATE*) [13], wheat (*TaMATE1*) [14], rye (*ScFRDL2*) [15], maize (*ZmMATE1*) [16], and rice (*OsFRDL4*) [17].

Al tolerance is quantitatively inherited in Arabidopsis [18], maize [19–21], and rice [22–26]. Evidences for internal Al detoxification as a complementary Al tolerance mechanism have been supported by the identification of genes encoding ATP binding cassette transporters in Arabidopsis (*ALS1* and *ALS3*) [27, 28] and rice (*STAR1*, *STAR2*[29]; *OsALS1*[30]). Additionally, Nramp aluminum transporter 1 (Nrat1) is an aluminum transporter localized on the plasma membrane, which is required in the initial steps of internal Al detoxification in rice [31]. The expression of many genes involved in Al tolerance is regulated by a Cys2-His2 zinc finger transcription factor in Arabidopsis (STOP1) [32, 33] and in rice (ART1) [34]. In fact, *ART1*, *STAR2* and *Nrat1* co-localized with Al tolerance QTLs in rice [26], indicating that QTL mapping is a useful tool to help in the validation of candidate Al tolerance genes.

Al tolerance is a complex trait in maize [35–37], with possible involvement of multiple genes and physiological mechanisms. One such mechanism is root citrate release [38, 39], although other mechanisms are likely to contribute to maize Al tolerance [40]. However, it is noteworthy that only a few QTLs [19–21] and candidate genes [16, 41, 42] have been reported for Al tolerance on this crop. Combining association and linkage mapping, four candidate genes were associated with Al tolerance in maize, *Zea mays Alt*_*SB*_-like (*ZmASL*), *ZmALMT2*, malic enzyme and S-adenosyl-L-homocysteinase [41]. However, functional characterization of *ZmALMT2* did not support a role in maize Al tolerance [43]. Two maize homologs of sorghum *SbMATE*, *ZmMATE1* and *ZmMATE2*, co-localized with Al tolerance QTLs [16] that were consistently detected in other QTL studies [19, 20]. *ZmMATE2* was not conclusively implicated in Al tolerance, but *ZmMATE1* is up-regulated by Al and encodes a membrane transporter responsible for Al-activated citrate exudation in root apices of Al-tolerant maize lines [16]. Recently, three copies of *ZmMATE1* were associated with high expression of this gene, which was proposed to underlie a major Al tolerance QTL mapped on maize chromosome 6 [42].

Here we performed a marker-trait association study with unprecedented marker density generated by genotyping-by-sequencing (GBS) [44] to provide a detailed description of the genetic complexity underlying maize Al tolerance. Five QTLs highly associated with Al tolerance were identified with 54,455 single nucleotide polymorphisms (SNPs). Based on our results, *ZmNrat1*, which encodes a protein sharing high amino acid sequence identity with the rice Al^3+^ transporter, Nrat1, is a candidate gene for further Al tolerance studies in maize. We also validated a major Al tolerance QTL, *qALT6*, which was able to improve Al tolerance in maize NILs. Finally, a novel Al tolerance source in maize based on *ZmMATE1* was identified, which does not rely on copy number variation to maintain high levels of *ZmMATE1* expression.

## Results

### Multiple genomic regions are associated with Al tolerance in maize

Significant genotypic differences for relative net root growth (RNRG) were detected in the RIL population, which was used to assess Al tolerance. Net root growth in control conditions (NRG_c) was also assessed as a measurement of intrinsic differences in root growth that are not necessarily related to Al tolerance (Table 1). The heritability estimates based on family means were 0.94 for RNRG and 0.92 for NRG_c, with a coefficient of variation close to 10% for both traits (Table 1).

**Table 1:** **Analyses of variance for relative net root growth and for net root growth in nutrient solution without Al in the recombinant inbred lines population**

Source of variation	DF	MS	
		RNRG	NRG_c
Genotypes	117	562.80**	864.00**
Residual	118	36.13	67.30
Total	235		
Coefficient of experimental variation (CVe%)		10.31	8.27
Coefficient of genetic variation (CVg%)		27.84	20.12
Heritability		0.94	0.92
CVg/CVe		2.70	2.43

Relative net root growth: RNRG; Net root growth in nutrient solution without Al: NRG_c.

DF: degrees of freedom; MS: mean square; **significant at *p* < 0.01.

Of the 458,255 SNPs generated with GBS, 54,455 SNPs were selected after imputing and filtering process (Additional file 1: Table S1). The imputation procedure improved by 43% the global number of SNPs, when compared with the selection of markers with less than 20% of missing data without imputing. Additionally, for chromosome 10, reducing MAF from 0.4 to 0.3 increased the number of SNPs from 694 to 2,803, indicating a significant segregation distortion of GBS-based markers on this chromosome.

**Figure 1:**
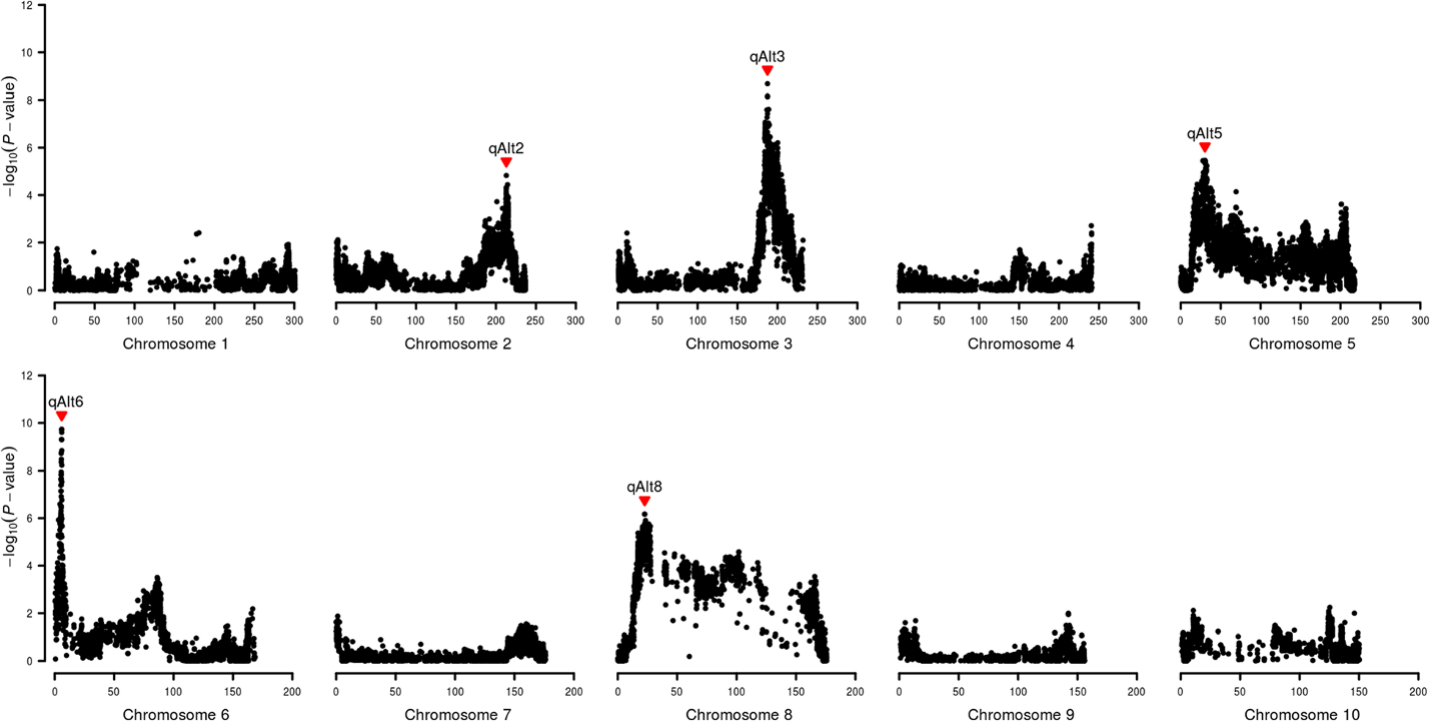
**Al tolerance QTLs detected using a multiple regression model.** QTLs were assigned as significant at *p* < 0.001.

The QTL mapping approach detected five genomic regions significantly associated with Al tolerance on maize chromosomes 2, 3, 5, 6, and 8. Together, these QTLs explained approximately 63% of the total phenotypic variance for RNRG (Table 2 and Figure 1). Epistatic interactions were not detected with the current population size. Three candidate genes for Al tolerance previously described in maize, *ZmMATE1*, *ZmMATE2*[16] and *ZmASL*[41] were genetically mapped to their predicted physical position based on anchored flanking SNPs. Two major Al tolerance QTLs were found on chromosomes 3 (*qALT3*) and 6 (*qALT6*), which individually explained ~27 and 30% of the variation in RNRG, respectively (Table 2). All the alleles increasing Al tolerance were donated by the Al-tolerant parent. Context sequences and SNP markers flanking each QTL were provided for approximately 200 bp windows defined based on the SNP markers with the highest -log_10_ (*P*-value) for association with Al tolerance (Additional file 2: Table S2).

**Table 2:** **QTLs identified through a multi-SNP regression model fitted with relative net root growth (RNRG) data**

QTL	Chr	SNP_ID	Position (Mbp)	-log _10_ ( ***P*** )	Effect	***R***_***P***_^***2***^ (%)	CI (Mbp)
*qALT2*	2	S2_212940514	212.94	4.83	9.14	15.47	208.20 - 215.41
*qALT3*	3	S3_187460236	187.46	8.68	13.11	27.51	186.73 - 188.90
*qALT5*	5	S5_30301926	30.30	5.46	9.82	17.56	18.70 - 40.10
*qALT6*	6	ZmMATE1	5.86	9.74	14.21	30.54	5.44 - 5.96
*qALT8*	8	S8_22681622	22.68	6.17	10.43	19.86	17.06 - 27.64
***R***_***T***_^***2***^ ***(%)***						62.78	

Chr: maize chromosome; SNP_ID indicates the chromosome followed by the physical position in bp; ZmMATE1 is the sequence-tagged site marker developed within *ZmMATE1*.

The position of each QTL was determined in Mbp (Mega base pairs) as the highest -log_10_ (*P*-value).

*R*_*P*_^*2*^(%) is the percentage of the phenotypic variance explained by each QTL and *R*_*T*_^*2*^(%) is the percentage of the phenotypic variance explained by the full model, including all significant QTLs.

CI is the 95% confidence interval of the QTL position.

Aluminum tolerance has been traditionally phenotyped based on the degree of root growth inhibition in Al-treated plants, relative to a -Al control, to remove possible confounding effects caused by intrinsic differences in root growth. To confirm that Al tolerance QTLs were not artifacts caused by variation in root growth unrelated to Al tolerance, QTLs were also mapped for NRG_c. Three genomic regions on chromosomes 1, 3, and 6 were associated with NRG_c, explaining approximately 57% of the total phenotypic variance (Table 3). Only the root growth QTL on chromosome 3 (*qNRG_c3*) was located near the Al tolerance QTL *qALT3*, but with opposite effects, indicating that for this QTL, selection to improve Al tolerance may cause a reduction in root length. Nevertheless, the other Al tolerance QTLs were not affected by root growth in the absence of Al.

**Table 3:** **QTLs identified through a multi-SNP regression model fitted with net root growth in nutrient solution without aluminum (NRG_c) data**

QTL	Chr	SNP_ID	Position (Mbp)	-log _10_ ( ***P*** )	Effect	***R***_***P***_^***2***^ (%)	CI (Mbp)
*qNRG_c1*	1	S1_69700844	69.70	7.98	−15.97	25.06	67.25 - 71.15
*qNRG_c3*	3	S3_195644732	195.64	10.50	−18.97	32.18	187.71 - 195.96
*qNRG_c6*	6	S6_158261558	158.26	10.20	−18.46	31.35	156.99 - 158.45
***R***_***T***_^***2***^ ***(%)***						56.88	

Chr: maize chromosome; SNP_ID indicate the chromosome and the physical position in bp.

The position of each QTL was determined in Mbp (Mega base pairs) as the highest –log_10_ (*P*-value).

*R*_*P*_^*2*^ *(%)* is the percentage of the phenotypic variance explained by each QTL and *R*_*T*_^*2*^ *(%)* is the percentage of the phenotypic variance explained by the full model, including all significant QTLs.

CI is the 95% confidence interval of the QTL position.

### Candidate genes on *qALT5*

**Figure 2:**
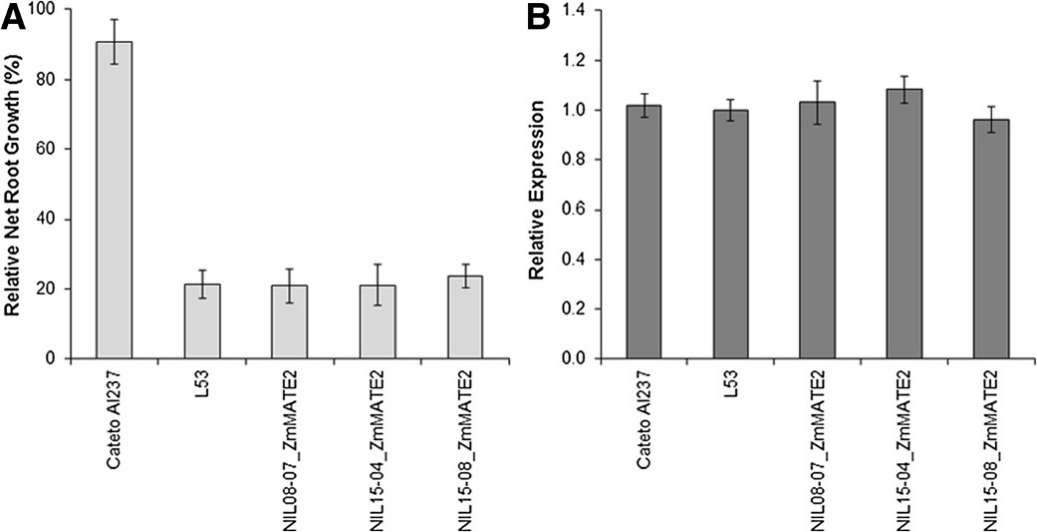
**Near-isogenic lines introgressed with** ***ZmMATE2*** **, using Cateto Al237 and L53 as the donor and recurrent parents, respectively. A)** Aluminum tolerance measured as relative seminal root growth after five days under {39} μM Al^3+^. **B)** *ZmMATE2* relative expression evaluated in root tips after 6 hours of treatment with {39} μM Al^3+^. Expression of L53 was used as calibrator. Error bars indicate standard deviation.

*qALT5* explained 17.6% of the phenotypic variance for RNRG and spanned a genomic region from 18.70 to 40.10 Mbp on chromosome 5. Based on the current annotation of the B73 RefGen_v2 genome sequence (5b.60), 432 gene models are predicted in this region, of which 30 were covered by at least one SNP significantly associated with Al tolerance (Additional file 3: Table S3). Among these genes, we selected *ZmMATE2* predicted at 20.60 Mbp, which was previously characterized as a membrane transporter and putatively associated with Al tolerance in maize [16]. Maize NILs on the Al sensitive background belonging to L53 introgressed with *ZmMATE2* were as Al-sensitive as was L53 (Figure 2a), and showed similar *ZmMATE2* expression levels to the parents (Figure 2b). *ZmMATE2* was not differentially expressed between the parental lines (Figure 2b), as reported by Maron *et al.*[16]. However, when the expression pattern of *ZmMATE2* was investigated in the RILs, one expression QTL (eQTL) explaining 19.9% of the variance for *ZmMATE2* expression was revealed at 204.66 Mbp on chromosome 3 (Additional file 4: Figure S1a), a position different from the target gene on chromosome 5.

**Figure 3:**
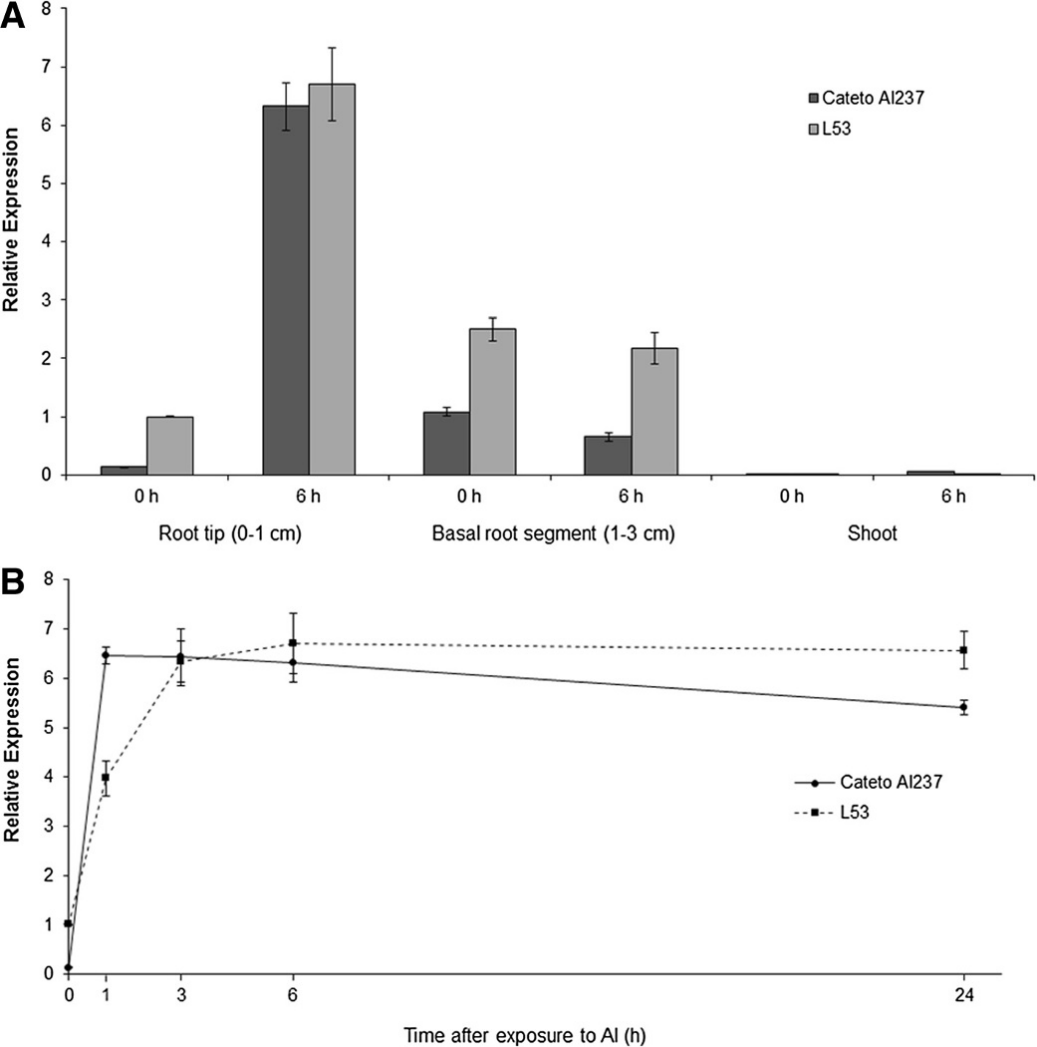
**Spatial and temporal expression profiles of** ***ZmNrat1*** **in two contrasting maize lines for Al tolerance. A)** Expression of *ZmNrat1* in different tissues under controlled conditions, and after 6 hours at {39} μM Al^3+^. **B)** Time course of *ZmNrat1* expression following treatment of root tips with {39} μM Al^3+^. Expression of L53 under controlled condition (−Al) was used as calibrator. Error bars indicate standard deviation.

Interestingly, another peak was detected close to *qALT5*, at 69.30 Mbp, but it was not significant in the multi-SNP regression model (Figure 1). In the vicinity of this putative peak, we identified the predicted gene GRMZM2G168747 at 74.61 Mbp, sharing 83% of amino acid sequence identity to rice Nrat1. The tissue specificity of *ZmNrat1* was then evaluated after 6 hours of Al exposure in both parental lines (Figure 3a). The results demonstrate that *ZmNrat1* is highly induced by Al in root tips, moderately expressed in basal root segments (1 to 3 cm above root tip) and is not expressed in shoots. A detailed time course response to Al treatment of root tips revealed that *ZmNrat1* was up-regulated by Al at very early stages in the tolerant line, reaching the maximum level after 1 hour of Al treatment, whereas in the Al-sensitive line the highest expression was achieved 3 hours after exposure to Al (Figure 3b).

### Candidate genes on *qALT6*

A highly significant QTL region was detected from 5.44 to 5.96 Mbp (*qALT6*), explaining a large proportion of the variation in Al tolerance. This genomic region encompassed *ZmMATE1* although none of the 54,455 SNPs tagged this candidate gene. Instead, a sequence-tagged site (STS) marker generated within *ZmMATE1* had the highest –log(*P-*value) for RNRG (Table 2). A single eQTL, explaining 70.9% of the variance for *ZmMATE1* expression, overlapped the *qALT6* region where *ZmMATE1* lies on chromosome 6 (Additional file 4: Figure S1b). This confirms previous results obtained at much lower SNP density, i.e. 1,894 SNPs on chromosome 6 [42].

**Figure 4:**
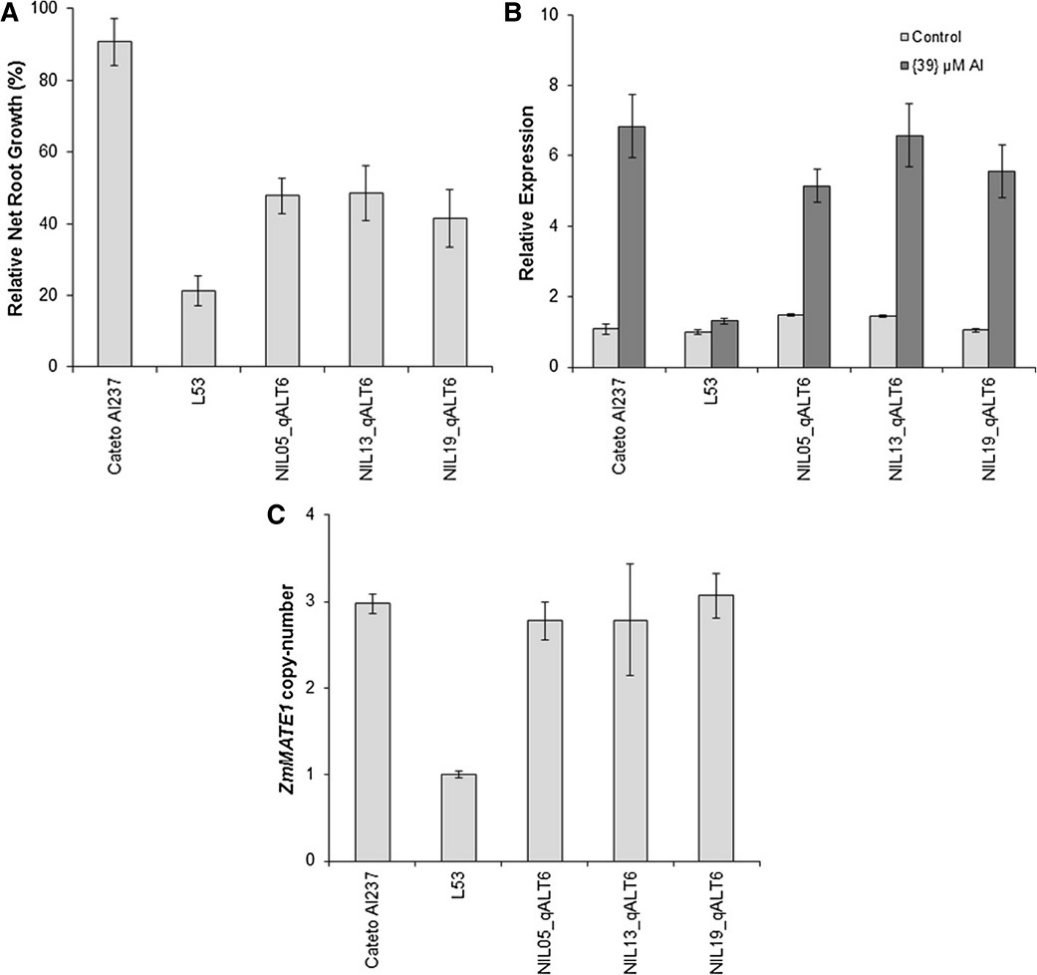
**Near-isogenic lines introgressed with** ***qALT6*** **, using Cateto Al237 and L53 as the donor and recurrent parents, respectively. A)** Aluminum tolerance measured as relative seminal root growth after five days under {39} μM Al^3+^. **B)** *ZmMATE1* relative expression evaluated in root tips after 6 hours of treatment with {39} μM Al^3+^ (dark gray) and under control condition (light gray). Expression of L53 under controlled condition (−Al) was used as calibrator. **C)** *ZmMATE1* copy-number estimated based on qPCR. Error bars indicate standard deviation.

The Cateto Al237 introgression region in the *qALT6*-NILs was estimated as 10 Mbp, extending from positions 3.4 to 13.4 Mbp on chromosome 6. This region includes the STS marker for *ZmMATE1* at position 5.86 Mbp, which showed the highest –log(*P-*value) with RNRG within *qALT6*. The *qALT6*-NIL harboring the Cateto Al237 allele showed a two-fold increase in Al tolerance when compared to the L53 counterpart (Figure 4a). The former exhibited also high *ZmMATE1* expression (Figure 4b) and three copies of *ZmMATE1*, similarly to the donor parent Cateto Al237 (Figure 4c).

### Phylogeny of MATE members in maize

Forty-three predicted MATE members found in the maize genome were clustered in two major groups, one of them comprising citrate transporters associated with Al tolerance in different plant species that included five maize MATEs (Additional file 5: Figure S2). These five predicted maize MATE proteins shared between 20 and 69% sequence identity to SbMATE, including ZmMATE1 and ZmASL, which was previously associated with Al tolerance [41] (Additional file 6: Table S4). Of the five candidate MATE members analyzed, only *ZmMATE1* was co-localized with Al tolerance QTLs mapped in this study.

### Identification of high *ZmMATE1*expression in Brazilian maize lines

**Figure 5:**
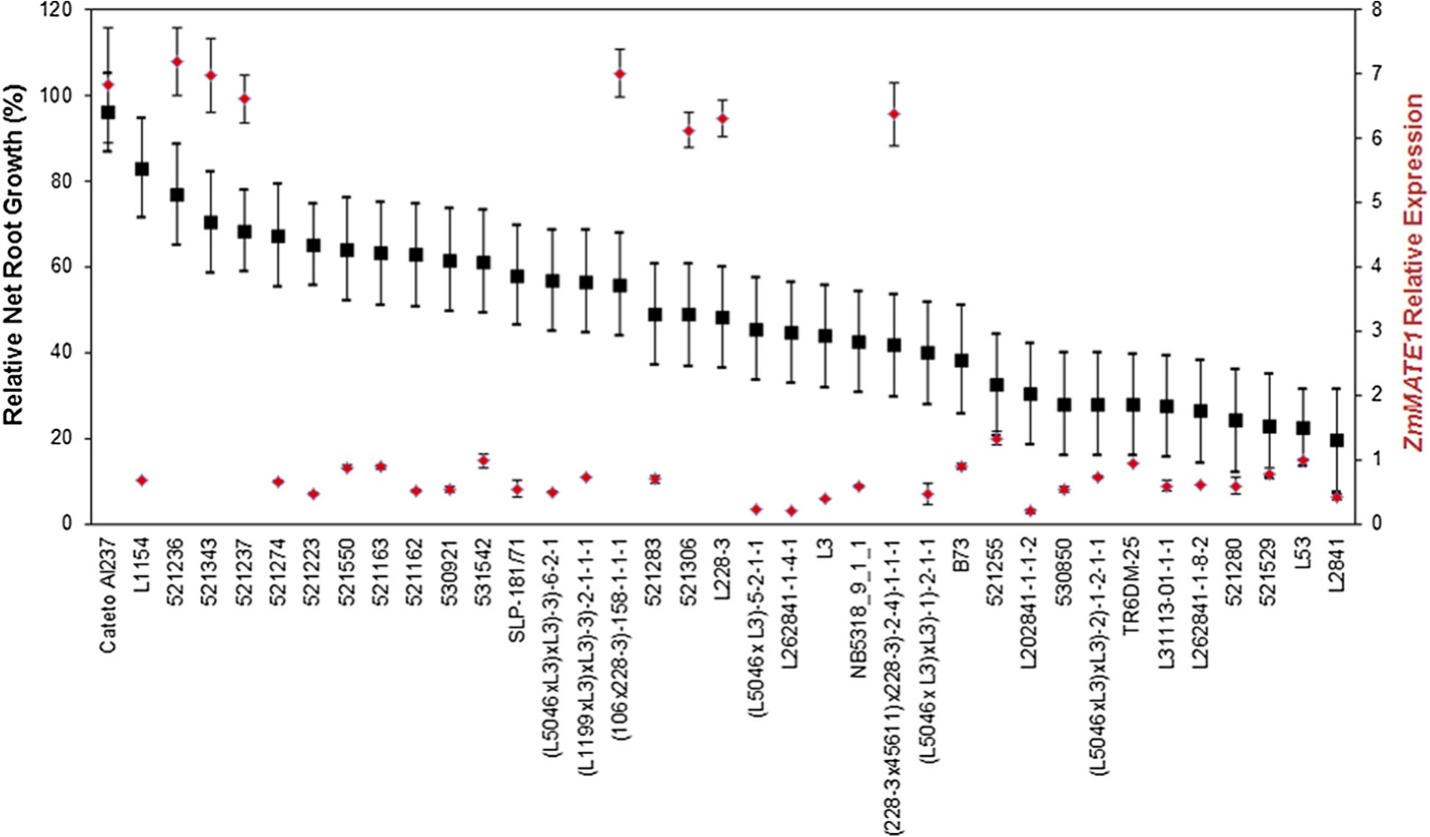
**Aluminum tolerance and** ***ZmMATE1*** **relative expression among 37 maize lines.** Aluminum tolerance measured as relative seminal root growth after five days under {39} μM Al^3+^ and *ZmMATE1* relative expression evaluated in root tips after 6 hours of treatment with {39} μM Al^3+^.

**Figure 6:**
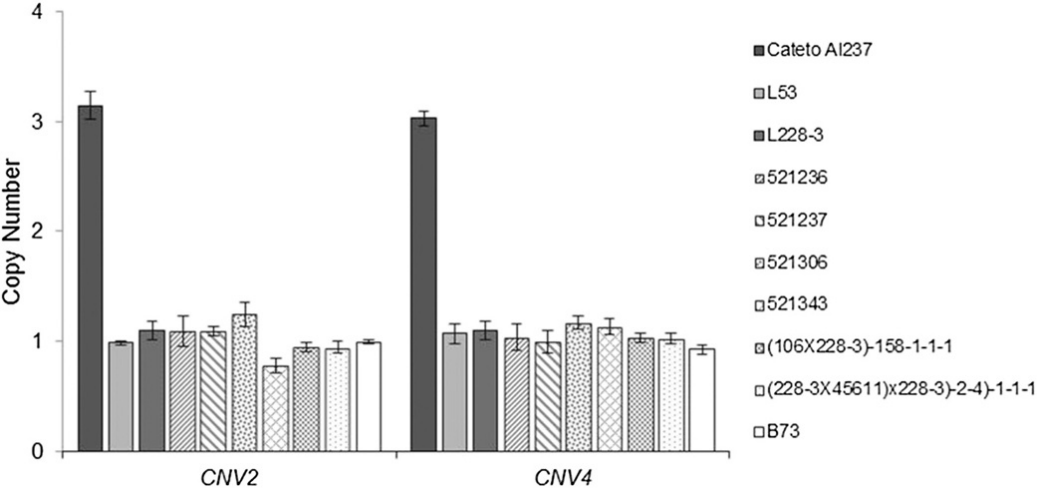
***ZmMATE1*** **copy-number estimate based on quantitative PCR in Cateto Al237, L53, L228-3, L228-3-derived lines (line designations starting with numbers), and B73.** The primers for CNV2 and CNV4 amplify a portion of exon 2 and 3′UTR region of *ZmMATE1*, respectively.

High *ZmMATE1* expression is associated with superior Al tolerance in maize RILs and NILs harboring the Cateto Al237 allele at *qALT6*. Thus, *ZmMATE1* expression pattern under Al stress was investigated in 36 maize lines derived from the Embrapa breeding program including the intermediately Al-tolerant, temperate maize line B73. The Embrapa elite lines showed a wide range of Al tolerance based on RNRG at {39} μM Al^3+^, ranging from Al-sensitive (RNRG < 30%) to highly Al-tolerant (RNRG > 80%) (Figure 5). The broad sense heritability estimate for Al tolerance was 0.95, with an accuracy of 0.97. In addition to the highly tolerant Cateto Al237, seven lines in the panel showed high *ZmMATE1* expression (Figure 5). These were L228-3 as well as six elite lines derived from L228-3, all of which are intermediately Al-tolerant. Interestingly, L228-3 and its derived elite lines did not carry the three copies of *ZmMATE1* reported for Cateto Al237 (Figure 6). The presence of a single copy of *ZmMATE1* in these maize lines was indicated by two qPCR assays, CNV2 and CNV4, amplifying a fragment of the second exon and the 3′UTR region of *ZmMATE1*, respectively [42]. Thus, high expression of *ZmMATE1* in these lines is likely mediated by a mechanism other than copy-number variation as proposed for Cateto Al237 [42].

## Discussion

The genetic complexity of Al tolerance in maize was dissected using a large number of markers provided by next-generation sequencing in a RIL population derived from two highly contrasting maize lines. For QTL mapping, a procedure similar to multiple interval mapping was implemented, based on a multi-SNP regression model, which accommodated multiple QTLs simultaneously.

Five genomic regions were found to be highly associated with Al tolerance as measured by RNRG, including the novel QTL, *qALT3*. We also mapped a large-effect QTL for root growth under control (−Al) condition that was relatively close to *qALT3* (*qNRG_c3*, at 195.64 Mbp on chromosome 3, Table 3). QTLs associated with root length were also previously mapped to a region surrounding *qALT3* (171–179 Mbp on chromosome 3) [45, 46]. Moreover, this region was flanked by two consensus QTLs, Rt-7 and Rt-8, which have been implicated in the control of several root traits in different maize populations [47]. Thus, although *qALT3* does not overlap with *qNRG_c3*, further use of this Al-tolerance QTL in maize breeding programs may lead to the introgression of undesirable alleles for root length due to linkage drag.

The other four Al tolerance QTLs coincided with regions previously reported to be related to maize Al tolerance. The RFLP markers umc122 and umc49, located at 209.5 and 222.5 Mbp on chromosome 2, respectively, were associated with Al tolerance in five different maize crosses [48]. Also, an Al tolerance QTL was mapped in a genomic region equivalent to 198.8 - 211.2 Mbp on maize chromosome 2 [20]. Despite the low saturation of markers used in these studies, it is plausible that these regions could be overlapping to *qALT2* (211.2 - 215.4 Mbp). Similarly, the Al tolerance QTL identified on chromosome 8 by Ninamango-Cárdenas *et al.*[20] is likely to correspond to *qALT8*.

The QTL, *qALT5,* reported here, was coincident with an Al tolerance QTL mapped on maize chromosome 5, which co-localized with *ZmMATE2*[16]. This candidate gene was previously characterized as a putative anion transporter that was however unable to mediate citrate efflux [16]. This is consistent with the phylogenetic analysis where ZmMATE2 was not included in the major group composed of all MATEs functionally identified as citrate transporters in other plants (Additional file 5: Figure S2). To test the hypothesis raised by Maron *et al.*[16] that the Al-sensitive line, L53, would carry positive regulatory factors for *ZmMATE2* expression, we mapped a *trans*-eQTL controlling part of *ZmMATE2* expression on chromosome 3. Our data confirmed that maize NILs with the *ZmMATE2* allele from Cateto Al237 in the genetic background of L53 showed the same *ZmMATE2* expression level as the parental lines. Additionally, these maize NILs were as Al-sensitive as the sensitive recurrent line, L53. Thus, *ZmMATE2* was constitutively expressed, independently of the source of *trans*-acting factors, either L53 or Cateto Al237. As ZmMATE2 amino acid sequences from Cateto Al237 and L53 were identical [16], we can infer that *ZmMATE2* is not the candidate gene controlling Al tolerance on *qALT5*, at least in our maize germplasm.

Outside of the *qALT5* confidence interval but near a putative QTL peak, we identified a maize homologue to rice *Nrat1*, named here as *ZmNrat1. OsNrat1* encodes an Nramp aluminum transporter involved in intracellular Al^3+^ uptake in rice [31]. In our study, *ZmNrat1* was up-regulated by Al primarily in root tips, following an expression pattern similar to that observed for the rice *OsNrat1*[31]. However, the main difference found between the contrasting maize lines for Al tolerance was the early Al-induction of *ZmNrat1* in the Al-tolerant Cateto Al237. A previous report based on microarray analysis indicated that *ZmNrat1* was also induced by Al in root tips of the Al-tolerant Cateto 100–6 after 2, 6 and 24 hours of Al treatment and only after 6 and 24 hours in the Al-sensitive line (L53) [49]. Thus, these studies consistently showed that *ZmNrat1* is rapidly induced by Al in tolerant maize lines (Cateto100-6 and Cateto Al237), whereas induction in the Al-sensitive line, L53, is delayed. Functional evidence in rice knockout mutants supported a role for *Nrat1* in Al tolerance [31]. In addition, genomic regions surrounding this candidate gene on rice chromosome 2 were also shown to contribute to natural variation for Al tolerance in a bi-parental QTL mapping [23] and in the *aus* subpopulation in a genome-wide association study [26]. Nevertheless, the *Nrat1* QTL region explained a low percentage of Al tolerance in a backcross introgression line population derived from *temperate japonica* x *aus*[23] and no QTL in this region was reported in a cross between *tropical japonica* and *indica*[26], suggesting that the effect of this QTL is dependent on the genetic background. The non-significance of the QTL peak spanning the *ZmNrat1* region may be explained by: i) the presence of a significant QTL (*qALT5*) close to this candidate gene that may have retained the genetic variance of this surrounding region; and ii) the reduced population size, which limits the power to resolve two closely linked QTLs. However, the evidence presented here warrants further investigation on the involvement of *ZmNrat1* on the internal Al detoxification mechanism in maize.

The major Al tolerance QTL on chromosome 6 (*qALT6*) was also identified in other QTL studies [16, 19, 20]. Within this QTL region, we mapped *ZmMATE1* as well as a major eQTL controlling its expression. The overexpression of *ZmMATE1* was shown previously to improve Al tolerance in transgenic Arabidopsis [16]. Here we demonstrate that the high expression of *ZmMATE1* almost doubled the Al tolerance of maize NILs when *qALT6* was introgressed into an Al-sensitive line. These NILs harbored three copies of *ZmMATE1*, confirming that the functional allele derived from Cateto Al237 was transferred. Interestingly, the expression pattern of *ZmMATE1* in maize NILs was quite different from *SbMATE* in sorghum NILs, which was down-regulated when different alleles were introgressed into a common, Al-sensitive background [50]. This reduction in *SbMATE* expression was closely correlated with a decrease in Al tolerance, suggesting an incomplete transfer of regulatory factors acting in *trans* on *SbMATE*[50]. Marker-assisted introgression of *SbMATE* would be dependent on the genetic background and may require additional genomic regions carrying accessory factors necessary for the full expression of *SbMATE*. In maize, introgression of *qALT6* seems to be enough for reaching full expression of *ZmMATE1*. Thus, our results, together with those presented in previous reports [16, 42], strongly support that *ZmMATE1* is the gene underlying *qALT6*, which enhances Al tolerance through its up-regulation under Al stress.

Cateto is a group of maize landraces from South America that were extensively adopted by native Indians and were also used as commercial varieties or in local hybrid programs [51]. Cateto lines were identified as highly Al-tolerant under field conditions [52] and in nutrient solution [53, 54] in the early 1980’s. Since then, Cateto lines have been considered as major sources of Al tolerance in maize. Three Cateto lines have been the subject of detailed genetic, physiological, and molecular studies of Al tolerance: Cateto Al237 [16, 20, 42, 49], Cat100-6 [19, 42, 49, 55, 56], and Cateto Colombia [39, 40]. The high Al tolerance of these lines in nutrient solution was mainly associated with an Al-exclusion mechanism mediated by Al-activated citrate exudation in root tips [16, 39, 40, 42]. Among those, only the native Brazilian lines, Cateto Al237 and Cat100-6, were confirmed to show high *ZmMATE1* expression controlled by three-copies of this gene, which is considered to be the major molecular mechanism underlying citrate exudation in those lines [42]. As Cateto landraces were widely cultivated by natives and early farmers in South America [51], it is reasonable to expect that their extreme Al tolerance accounts for their superior adaptation to acid soils. However, these landraces carry undesirable traits, reducing their potential for direct use in commercial maize breeding programs [57]. Thus, the introgression of Al tolerance alleles from native races into maize elite lines is an important target to improve maize stability in tropical soils.

A further characterization of tropical maize lines revealed that L228-3 and its derived elite lines also presented high *ZmMATE1* expression. However, in contrast to Cateto Al237 and Cat100-6 lines that carry this gene in triplicate [42], only a single copy of *ZmMATE1* was found in L228-3 and its derived lines. L228-3 is the main founder of the dent heterotic group from the Embrapa maize breeding program [58], which was derived from the open pollinated variety BR106, as a result of 13 selection cycles in the Tuxpeño Composite [59]. Elite dent lines extracted from BR106 were used to develop commercial hybrids widely cultivated in Brazilian Cerrado, including BR201, a double cross hybrid that occupied up to 14% of the Brazilian seed market from 1989 to 1993 [60]. We can infer that, in L228-3, a different molecular mechanism to copy number variation (CNV) would enhance *ZmMATE1* expression under Al stress, conferring an important advantage to acid soils adaptation.

## Conclusions

The high *ZmMATE1* expression controlled either by CNV or by another molecular mechanism that underlies *qALT6*, in addition to significantly improving Al tolerance in maize, is rare and restricted to a few South American landraces, mainly from Brazil and Colombia. Thus, we may expect that a large proportion of modern maize lines that range from Al sensitive to intermediate in Al tolerance could be improved for Al tolerance via marker-assisted introgression of this genomic region.

## Methods

### Genetic stocks

A population of 118 recombinant inbred lines (RILs) in S7, derived from a cross between Cateto Al237 and L53, was used for QTL mapping. Cateto Al237 and L53 have been characterized as highly Al tolerant and Al-sensitive, respectively.

### Assessment of aluminum tolerance in nutrient solution

Experiments were performed in a growth chamber at 27/20°C day/night temperatures, with light intensity of 330 μmol photons m^–2^ s^–1^ and a 12-h photoperiod. Maize seeds were surface-sterilized with 0.5% (w/v) NaOCl for 5 minutes, thoroughly rinsed in deionized water and germinated for four days on moistened germination paper rolls. Seedlings were transferred to polyethylene cups placed into containers filled with 8.5 l of nutrient solution at pH 4.0, according to Magnavaca *et al.*[35], under continuous aeration, and acclimatized in full nutrient solution for 24 h. After the initial root length (IRL) was measured, +/− Al treatments were imposed by replacing the nutrient solution with nutrient solution of identical composition but with or without {39} μM Al^3+^ activity supplied as KAl(SO_4_)_2_. Free Al^3+^ activities (number in brackets) were calculated using the GEOCHEM-EZ speciation software [61]. The solution pH was adjusted to pH 4.0. After five days, the final root length (FRL) of each plant was measured. Net root growth (NRG) was calculated as NRG = FRL - IRL, either under Al treatment (NRG_Al) or under control condition, without Al (NRG_c). Finally, Al tolerance was expressed as Relative Net Root Growth (RNRG) estimated as RNRG = NRG_Al/NRG_c.

Experiments were performed in a complete randomized design with two replicates and seven plants per plot. Analysis of variance was performed using the Genes software [62].

### Molecular markers

Genomic DNA was isolated from leaf tissue using the CTAB protocol [63]. Sequence-tagged site (STS) markers for candidate genes were designed based on polymorphisms detected in the parents. *ZmMATE1* was genotyped with a 129-bp indel amplified with the primers 5′-CCGGATGTTTGCTGGATTTT-3′ and 5′-TGGCCAAATCGACCATGATT-3′, and resolved on 1% (w/v) agarose gel stained with ethidium bromide. *ZmMATE2* was mapped with a 10-bp indel detected with the fluorescently labeled primers, 5′-GCAGTTCGTACGTAGTGGTG-3′ and 5′-AGTACGTAGCTAGGCGATGC-3′, in the ABI3100 genetic analyzer (Applied Biosystems). For *ZmASL*, the primers 5′-CCGGCACAGCAGTATCAAC-3′ and 5′-TTGCTTTCCCCGATAGAGAA-3′ were designed based on reference sequences obtained from the Maize Assembled Genomic Island (MAGI) Database [64], which detected a 25-bp indel in the parents. *ZmNrat1* [MaizeGDB: GRMZM2G069198] was genotyped using a Cleaved Amplified Polymorphic Sequence (CAPs) marker based on a genomic fragment amplified with primers 5′-CGCGGAAACAGGAACCAAACCAAAA-3′ and 5′-CGGGTCTCTGCGTACCCCGA-3′, cleaved with *Hinf*I. The STS and CAPs markers described above were genotyped on 1% (w/v) agarose gels.

SNPs were genotyped using genotyping-by-sequencing as described in Elshire *et al.*[44]. SNPs were designated with the letter S followed by the chromosome number and the physical position along each chromosome. Missing data imputation was performed with the software Npute [65]. SNP filtering was performed using the TASSEL software version 3.0.83 [66] with a minimum minor allele frequency (MAF) threshold of 0.4, except for chromosome 10 that was filtered using a minimum MAF of 0.3, due to a high percentage of SNP markers showing segregation distortion.

### QTL mapping

The procedure used for QTL mapping was similar to multiple interval mapping [67], which allows fitting multiple QTL simultaneously with main effects and epistatic interactions. The high marker density provided by GBS precluded the need for estimating conditional probabilities of marker genotypes.

First, a single marker analysis was performed using a linear regression model. Markers associated with Al tolerance were declared considering a p < 0.001 threshold. Next, a multiple regression model was fitted by adding the most significant SNP to the model selected in the preceding step. This was done in a stepwise fashion until no more significant SNPs were found. Finally, we tested all pairwise epistatic interactions among SNPs that were kept in the model as well as between those and all other SNPs.

SNP effects, partial (*R*^*2*^_*P*_) and total (*R*^*2*^_*T*_) coefficients of determination were estimated from a multi-SNP regression model. *R*^*2*^_*T*_ was calculated from the full model including all SNP effects, whereas *R*^*2*^_*P*_ was estimated for each SNP as follows. For a given significant SNP, we estimated the sum of squares of residuals from the reduced model (*SSErm*) without the SNP effect and the sum of squares of residuals from the full model (*SSEfm*). *R*^*2*^_*P*_ was estimated as 1-(*SSEfm*/*SSErm*). All QTL mapping procedure was implemented in the software R [68].

Information of SNP markers flanking each QTL were obtained from Panzea (http://www.panzea.org/db/searches/webform/marker_search). Markers were selected in a window size of approximately 200 bp to the SNP associated with Al tolerance at highest -log_10_ (*P*-value).

### Development of NILs for the two major Al tolerance QTL

RIL-150 and RIL-84 were selected based on the presence of the Cateto Al237 allele at the Al tolerance QTLs, *qALT6* and *qALT5*, respectively. These RILs were crossed with the Al sensitive recurrent parent, L53, following two cycles of marker-assisted backcrosses and selfing until BC_2_F_3_ progeny fixed for the Al tolerance QTL were obtained. For *qALT6*, foreground selection was based on a STS marker for *ZmMATE1* and the SSR marker, umc1018, whereas for *qALT5* we used only the STS marker for *ZmMATE2*. Background selection was performed using ~30 simple sequence repeats (SSR) markers randomly distributed along the maize genome, which allowed for the selection of NILs with ~98% of the L53 genome. NILs for *qALT6* were genotyped-by-sequencing to evaluate the size of the Cateto Al237 introgression.

### Expression pattern of *ZmMATE1*, *ZmMATE2*and *ZmNrat1*

Temporal and spatial expression profiles of the candidate genes were obtained using quantitative real-time PCR (qPCR), after 6 hours of Al treatment. For this analyses, the first centimeter of root apices were sampled as previously described [16]. Expression of *ZmNrat1* was also assessed in a root segment extending from 1 to 3 cm from the root tip, which was designated as basal root segment, and in shoot tissues. The time-course profile for *ZmNrat1* expression in root tips was obtained after 1, 3, 6 and 24 hours of exposure to {39} μM Al^3+^, and compared to the control lacking Al. Total RNA was extracted using the RNeasy Plant Mini Kit (Qiagen) according to the manufacturer’s instructions. First-strand cDNA was synthesized using the High Capacity cDNA Reverse Transcription Kit (Life Technologies). *ZmMATE1* expression was determined using a custom-designed TaqMan assay consisting of forward primer 5′-CACCCGCTTAGCGTATTCCT-3′, reverse primer 5′-GCACCGCGATCCTCATGAT-3′ and probe 5′-TCTGAATGCGAGCCTCG-3′, with a pre-designed TaqMan assay for Eukaryotic 18S (Life Technologies) as endogenous control [42]. *ZmMATE2* expression was evaluated using a TaqMan assay according to Maron *et al.*[16]. The expression profile of *ZmNrat1* was conducted using the Fast SYBR® Green Master Mix (Life Technologies) with primers F: 5′-CGCGCTTCTGATCCAAACA-3′ and R: 5′-GCGAGATGCTTGCCTGTCTT-3′, with 18S RNA as endogenous control. qPCR reactions for *ZmMATE2* and *ZmNrat1* were conducted in an ABI 7500 Fast (Life Technologies). Each biological sample was composed by 21 plants and 3 technical replicates were adopted. Raw data were collected using the RQ Manager software (Life Technologies) and the relative expression levels were calculated using the ddCT method [69]. Relative expression data for *ZmMATE1* and *ZmMATE2* were used for expression QTL (eQTL) mapping.

### Copy number quantification for *ZmMATE1*

Copy number for *ZmMATE1* was estimated by qPCR using two primer pairs designed by Maron *et al.*[42] with the Fast SYBR® Green Master Mix in an ABI 7500 Fast (Life Technologies). The CNV2 assay was performed with primers F: 5′-CCAGGCTCGCATTCAGATG-3′and R: 5′-GCACCGCGATCCTCATG-3′, and CNV4 with F: 5′-TGTGAGTTTGGCGGATGTGT-3′ and R: 5′-TCACAATCTAGGCCAGTACAACAGA-3′, both using the actin gene as a single copy control [42]. After optimization, the reactions were performed using 6 ng of genomic DNA with 0.6 pmol and 0.3 pmol of primers for CNV2/CNV4 and the control, respectively. Three technical replicates were performed for each sample and assay. Relative quantification (RQ) was calculated by dividing the target sample quantity by the control sample quantity mean, then normalizing against the calibrator sample, L53, using the ddCT method [69].

### Phylogenetic studies of *MATE*genes in the maize genome

MATE proteins in the maize genome were identified by sequence similarity analysis using the *Sorghum bicolor* SbMATE amino acid sequence [GenBank: ABS89149.1] as query in the B73 RefGen_v2 filtered set (Maize Genome Sequencing Project, release 5b.60, http://www.maizesequence.org). The Peptide Homologs tool in Phytozome was used to confirm that all predicted maize MATEs were identified.

The amino acid sequences of maize MATEs as well as the MATE genes previously identified as Al tolerance genes in other crops, such as sorghum (*SbMATE*), Arabidopsis (*AtMATE*), rice (*OsFRDL4*), barley (*HvAACT1*), rye (*ScFRDL2*) and wheat (*TaMATE1*), were aligned using the Advanced M-COFFEE package available at T-COFFEE (http://www.tcoffee.org). The phylogenetic tree was constructed using maximum parsimony and 500 bootstraps with MEGA5 [70].

### Al tolerance in a maize panel

Thirty six tropical maize inbred lines developed by the Embrapa Maize and Sorghum (Sete Lagoas, Brazil) breeding program, as well as the temperate maize line B73, were evaluated for Al tolerance. Linear mixed model analysis was performed using an incomplete block design with common checks and three replicates, considering genotypes and blocks as random effects and experiment as a fixed factor. The experimental unit consisted of seven plants per genotype. Two maize lines, L53 and Cateto Al237, were used as common checks in each experiment. The linear mixed model analysis was performed in the software SELEGEN-REML/BLUP [71] according to the model: *y = Xt + Zg + Wb + e*, where: *y* is the vector of phenotypes; *t* is the vector of experimental effects (fixed) plus the overall mean; *g* is the vector of genotypic effects (random); *b* is the vector of block effects (random); and *e* is the error vector (random). X, Z and W represent the incidence matrices for the effects of *t*, *g* and *b*, respectively. The means and variances were structured and distributed as follows:


The genotypic values (μ + g) were used to characterize the Al tolerance of these lines, and the confidence intervals (CI) were estimated using the standard error of the predicted genotypic values (SEP), which corresponded to the squared root of predicted error variance (PEV), according to the expression: IC = (μ + g) ± t x SEP, where: t_(0.95)_ = 1.96 is the value of Student distribution at 95% of confidence interval. Non-overlapping confidence intervals indicate statistical differences of predicted genotypic effects in multiple comparisons.

## Electronic supplementary material

Additional file 1: Table S1: Genotyping-by-sequencing SNPs generated across the 10 maize chromosomes in the recombinant inbred line population. (DOCX 20 KB)

Additional file 2: Table S2: SNP markers flanking each Al tolerance QTL in a windows size of approximately 200 bp to the SNP associated with Al tolerance at highest *P-value*. (XLSX 30 KB)

Additional file 3: Table S3: Predicted genes from 18.70 to 40.10 Mbp on chromosome 5, comprising the *qALT5*. (XLSX 51 KB)

Additional file 4: Figure S1: Expression QTLs (eQTLs) of *ZmMATE2* and *ZmMATE1* on chromosomes 3 and 6, respectively. (TIFF 89 KB)

Additional file 5: Figure S2: Maximum likelihood phylogenetic tree of maize MATE members and MATE proteins characterized as citrate transporters in other plant species. Numbers in the nods indicate bootstrap values calculated using 500 resampling. (TIFF 108 KB)

Additional file 6: Table S4: Predicted maize MATE members clustered with citrate transporter from other plants, aminoacid sequence identity to SbMATE and the predicted physical position on the maize genome. (DOCX 19 KB)

## Abbreviations

Al: Aluminum
ALMT1: Aluminum-activated malate transporter
ALS: Aluminum sensitive
ART1: Al resistance transcription factor
BC2F3: Backcross 2 F3
bp: Base pair
CAPs: Cleaved amplified polymorphic sequence
CI: Confidence interval
CNV: Copy number variation
eQTL: Expression quantitative trait loci
FRL: Final root length
GBS: Genotyping-by-sequencing
HvAACT1: *Hordeum vulgare* aluminum-activated citrate transporter
indel: Insertion/deletion
IRL: Initial root length
MAF: Minor allele frequency
MAGI: Maize assembled genomic island
MATE: Multidrug and toxic compound extrusion
Mbp: Mega base pairs
μM: micro Molar
NIL: Near-isogenic line
Nramp: Natural resistance-associated macrophage protein
Nrat1: Nramp aluminum transporter
NRG_c: Net root growth in control conditions
OsFRDL4: *Oryza sativa* ferric reductase defective 4
PEV: Predicted error variance
qPCR: Quantitative real-time PCR
QTL: Quantitative trait loci
RIL: Recombinant inbred line
RNRG: Relative net root growth
SEP: Standard error of the predicted genotypic values
ScFRDL2: *Secale cereale* ferric reductase defective 2
SNP: Single nucleotide polymorphism
SSEfm: Sum of squares of residuals from the full model
SSErm: Sum of squares of residuals from the reduced model
SSR: Simple sequence repeats
STAR: Sensitive to Al rhizotoxicity
STOP1: Sensitive to proton rhizotoxicity 1
STS: Sequence-tagged site
w/v: Weight per volume
ZmASL: *Zea mays* AltSB-like.
