# Supplementary material for: Genetic dissection of Al tolerance QTLs in the maize genome by high density SNP scan
Source: BMC Genomics. 2014 Feb 24;15(1):153. doi: 10.1186/1471-2164-15-153 (PMC4007696; doi:10.1186/1471-2164-15-153)
Supplement: Supplementary file 1 — Additional file 1: Table S1: Genotyping-by-sequencing SNPs generated across the 10 maize chromosomes in the recombinant inbred line population. (DOCX 20 KB) [file 12864_2013_7015_MOESM1_ESM.docx]

Table S1. Genotyping-by-sequencing SNPs generated across the 10 maize chromosomes in the recombinant inbred line population

| **Chromosomes** | **Total SNPs** | **Imputed and Filtered SNPs*** |
| --- | --- | --- |
| 1 | 72,425 | 7,055 |
| 2 | 56,065 | 8,185 |
| 3 | 53,491 | 5,657 |
| 4 | 42,444 | 5,150 |
| 5 | 52,724 | 7,336 |
| 6 | 37,381 | 5,190 |
| 7 | 39,131 | 5,891 |
| 8 | 38,745 | 3,543 |
| 9 | 33,806 | 3,645 |
| 10 | 32,043 | 694/2,803^#^ |
| **Total** | **458,255** | **54,455** |

*Filtering criterion: minimum minor allele frequency (MAF) of 0.4, except for chromosome 10.

^#^Number of SNP markers selected using minimum MAF of 0.4 and 0.3, respectively.
