# Supplementary figures and images for: Genetic dissection of Al tolerance QTLs in the maize genome by high density SNP scan
Source: BMC Genomics. 2014 Feb 24;15(1):153. doi: 10.1186/1471-2164-15-153 (PMC4007696; doi:10.1186/1471-2164-15-153)

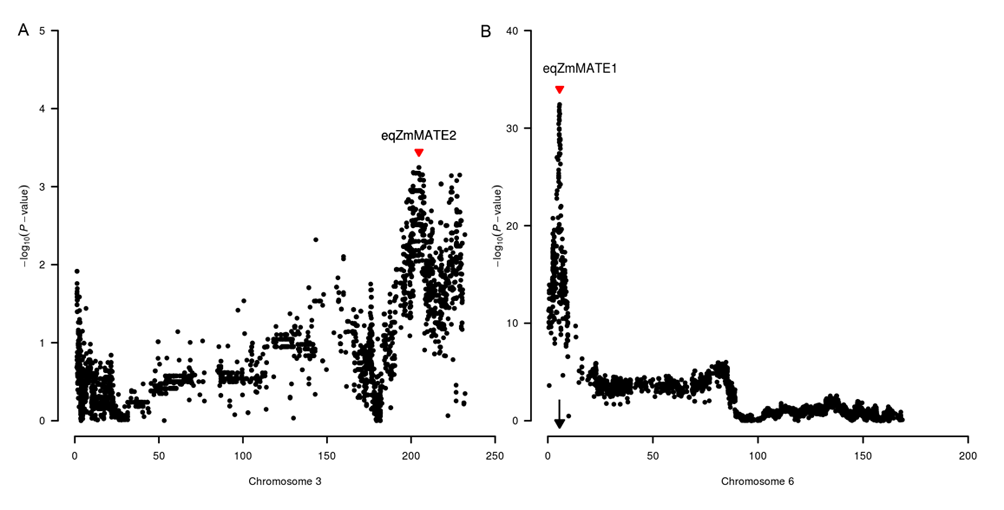

Supplement: Supplementary file 4 — Additional file 4: Figure S1: Expression QTLs (eQTLs) of ZmMATE2 and ZmMATE1 on chromosomes 3 and 6, respectively. (TIFF 89 KB) [file 12864_2013_7015_MOESM4_ESM.tiff]

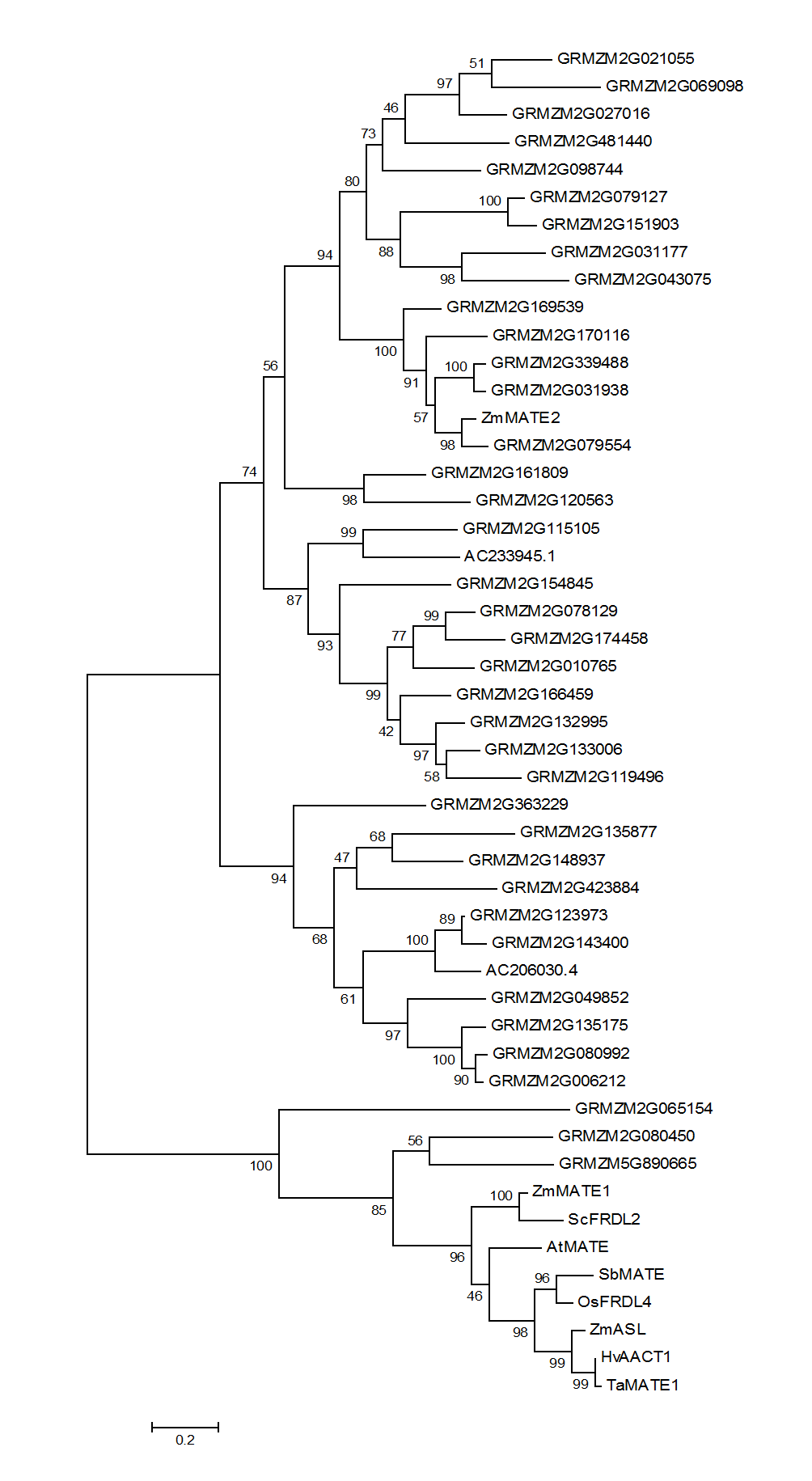

Supplement: Supplementary file 5 — Additional file 5: Figure S2: Maximum likelihood phylogenetic tree of maize MATE members and MATE proteins characterized as citrate transporters in other plant species. Numbers in the nods indicate bootstrap values calculated using 500 resampling. (TIFF 108 KB) [file 12864_2013_7015_MOESM5_ESM.tiff]
