# Supplementary material for: Genetic dissection of Al tolerance QTLs in the maize genome by high density SNP scan
Source: BMC Genomics. 2014 Feb 24;15(1):153. doi: 10.1186/1471-2164-15-153 (PMC4007696; doi:10.1186/1471-2164-15-153)
Supplement: Supplementary file 6 — Additional file 6: Table S4: Predicted maize MATE members clustered with citrate transporter from other plants, aminoacid sequence identity to SbMATE and the predicted physical position on the maize genome. (DOCX 19 KB) [file 12864_2013_7015_MOESM6_ESM.docx]

Table S4. Predicted maize MATE members clustered with citrate transporter from other plants, aminoacid sequence identity to SbMATE and the predicted physical position on the maize genome.

| **Gene ID** | **Gene Name** | **Identity (%)** | | **Chromosome** | **Position (Mbp)** |
| --- | --- | --- | --- | --- | --- |
| GRMZM2G163154 | *ZmALS* | 69.0 | 1 | | 27.10 |
| GRMZM5G870170 | *ZmMATE1* | 52.6 | 6 | | 5.87 |
| GRMZM2G080450 |  | 39.0 | 3 | | 57.08 |
| GRMZM5G890665 |  | 38.0 | 2 | | 152.91 |
| GRMZM2G065154 |  | 20.2 | 5 | | 71.70 |
